# P-2310. *Gammaproteobacteria* Load Responses in the Intestinal Microbiota of Patients Undergoing Hematopoietic Cell Transplantation Predicts Resistant Gram-Negative Rod Colonization

**DOI:** 10.1093/ofid/ofae631.2462

**Published:** 2025-01-29

**Authors:** Leanne Mortimer, Jaxon Sénéchal, Nicole Janusz, Amanda C Carroll, Austin Yan, Tamara Leite, Andrew Purssell, Natasha Kekre, Michael Kennah, C Arianne Buchan, Derek MacFadden

**Affiliations:** University of Ottawa, Ottawa, Ontario, Canada; Ottawa Hospital Research Institute, Ottawa, Ontario, Canada; University of Ottawa, Ottawa, Ontario, Canada; The Ottawa Hospital Research Institute, Ottawa, Ontario, Canada; University of Ottawa, Ottawa, Ontario, Canada; Ottawa Hospital Research Institute, Ottawa, Ontario, Canada; Ottawa Hospital Research Institute, Ottawa, Ontario, Canada; Ottawa Hospital Research Institute, Ottawa, Ontario, Canada; The Ottawa Hospital, Ottawa, Ontario, Canada; The Ottawa Hospital - General Campus, Ottawa, ON, Canada; The Ottawa Hospital Research Institute, Ottawa, Ontario, Canada

## Abstract

**Background:**

Hematopoietic cell transplant (HCT) patients are at high risk of Gram-negative rod (GNR) blood stream infections (BSIs) particularly before pre-engraftment. Globally, breakthrough infections with resistant GNRs (rGNRs) are increasing in HCT while rGNR epidemiology has become increasingly complex to predict. It’s recognized that individualized antimicrobial HCT therapy is needed but it is unclear what screening strategies are optimal to detect rGNR colonization. Single taxon dominance of certain *Gammaproteobacteria* (ɣ-bacteria) species in HCT intestinal microbiomes is a BSI risk factor where colonizing strains present prior to transplant are predominant aetiologies of GNR BSIs. In this study we sought to understand how ɣ-bacteria loads respond to HCT antimicrobial therapy.

**Figure:**
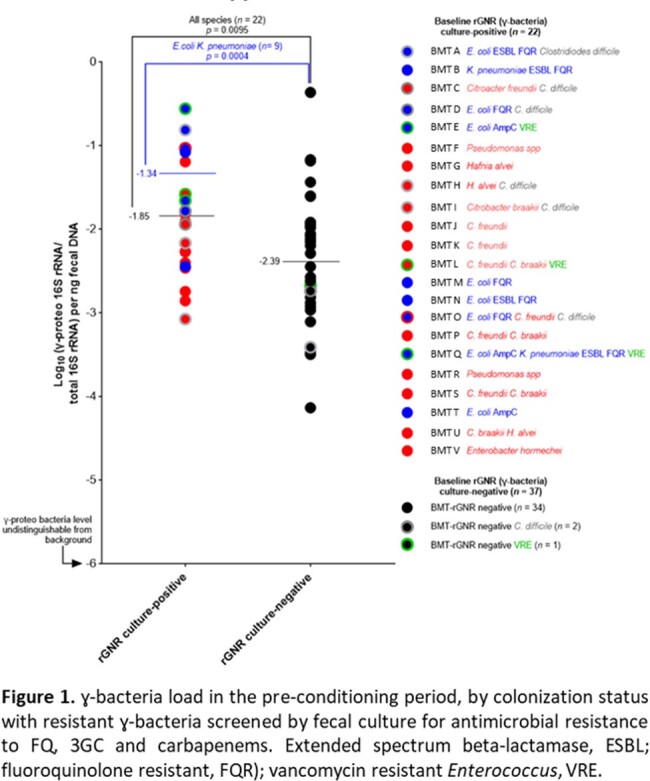

**Methods:**

We performed a prospective study of 60 adult patients undergoing HCT at The Ottawa Hospital from 2021-2022. A baseline fecal sample collected < 1 week before conditioning was screened by culture for colonization with rGNR to fluoroquinolones (FQ), 3rd generation cephalosporins (3GC) and carbapenems. Total- and ɣ-bacteria loads were measured by qPCR in baseline and serial specimens until engraftment, expressed as the Log_10_ (ɣ-bacteria 16S copies / total 16S copies) per ng fecal DNA. Ceftriaxone (CTX) and piperacillin-tazobactam (TZP) are used for prophylaxis and empiric FN therapy, respectively at our institution.

**Figure:**
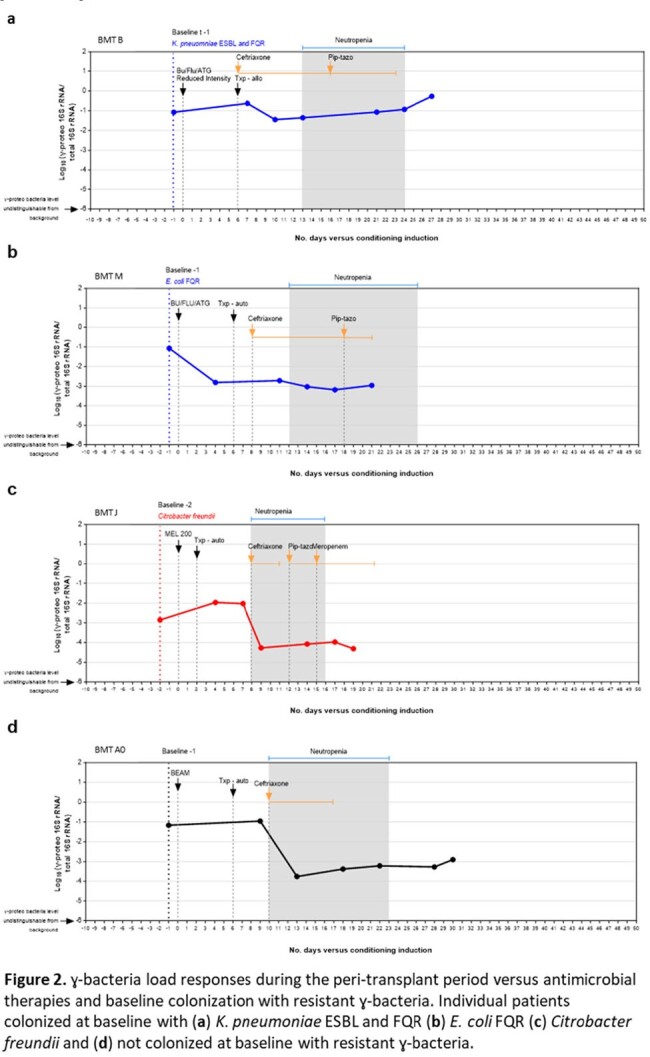

**Results:**

High ɣ-bacteria loads were associated with baseline rGNR colonization compared to no rGNR colonization (Log_10_ (-1.85) vs Log_10_ (-2.39), p < 0.0095) (Fig. 1). Highest loads occurred when resistant *Escherichia coli* or *Klebsiella pneumoniae* colonized the intestinal microbiota (Log_10_ (-1.34), p < 0.001). ɣ-bacteria loads were refractory to CTX and TZP therapy when colonizing *E. coli* or *K. pneumoniae* carried 3GC resistance (Fig. 2a). In contrast, ɣ-bacteria loads decreased > 2 Log_10_ in response to CTX and TZP if at baseline rGNRs were FQ resistant, expressed chromosomal AmpC, or if no rGNRs were detected (Fig. 2b, c, d).

**Conclusion:**

ɣ-bacteria load measurement during HCT may be useful for predicting colonization with rGNR strains and identifying patients at risk of GNR BSI.

**Disclosures:**

All Authors: No reported disclosures

